# The Role of Pancreatic Infiltrating Innate Immune Cells in Acute Pancreatitis

**DOI:** 10.7150/ijms.51618

**Published:** 2021-01-01

**Authors:** Cheng Peng, Zhiqiang Li, Xiao Yu

**Affiliations:** Department of Hepatopancreatobiliary Surgery, Third Xiangya Hospital, Central South University, Changsha 410013, Hunan, China.

**Keywords:** acute pancreatitis, immune cell, inflammation, macrophage, neutrophil

## Abstract

Acute pancreatitis (AP) is a leading cause of gastrointestinal-related hospital admissions with significant morbidity and mortality. Although the underlying pathophysiology of AP is rather complex, which greatly limits the treatment options, more and more studies have revealed that infiltrating immune cells play a critical role in the pathogenesis of AP and determine disease severity. Thus, immunomodulatory therapy targeting immune cells and related inflammatory mediators is expected to be a novel treatment modality for AP which may improve the prognosis of patients. Cells of the innate immune system, including macrophages, neutrophils, dendritic cells, and mast cells, represent the majority of infiltrating cells during AP. In this review, an overview of different populations of innate immune cells and their role during AP will be discussed, with a special focus on neutrophils and macrophages.

## Introduction

Acute pancreatitis (AP) is a leading cause of gastrointestinal-related hospital admissions 1. There are regional and ethnic differences in the etiology of AP, overall, gallstones and alcohol abuse are the two most common causes of AP, while hypertriglyceridemia ranks third place with an increasing trend. Other less common causes include hypercalcemia, cigarette smoking, drug reactions, genetic factors, etc. 2. Around 80% of cases of AP are mild AP (MAP) 3, with only interstitial changes of the pancreas, which can usually be relieved within 2 weeks, while patients with severe AP (SAP) are associated with persistent organ failure and multi-organ dysfunction die 4. Currently, it is generally accepted that AP initiates local inflammation and injury in the pancreas; then the inflammation is amplified due to the inflammatory cascade, which ultimately leads to systemic inflammatory response syndrome (SIRS), multiple organ dysfunction syndromes (MODS) or even multiple organ failure (MOF). However, due to an obscure pathophysiological mechanism, AP remains considerable incidence and mortality 5, 6. Therefore, it is essential to explore the pathogenesis of AP, especially the mechanisms of inflammation and immune response activation.

Immune responses are an integral part of the pathogenesis of pancreatitis 7. Evidence shows that the excessive systemic inflammation associated with AP is a consequence of uncontrolled or dysregulated activation of the immune system 8. In the early phase of AP, the pancreatic acinar cell injury occurs in an aseptic environment since the uninflamed pancreas is a sterile organ, which leads to proinflammatory mediators release, immune cells infiltration and sterile inflammation 7. Therefore, pathogen-associated molecular patterns (PAMPs) play no role in the recruitment and activation of immune cells in early AP 9. Instead, the necrotized pancreatic acinar cells release various kinds of danger-associated molecular patterns (DAMPs), including high-mobility group box protein 1 (HMGB1), self-DNA, and so on 10, 11. Subsequently, these DAMPs activate the pattern recognition receptors (PRRs) of infiltrating immune cells to produce more inflammatory mediators, which in turn promote more immune cell infiltration and aggravate inflammation 12.

**Figure 1:**
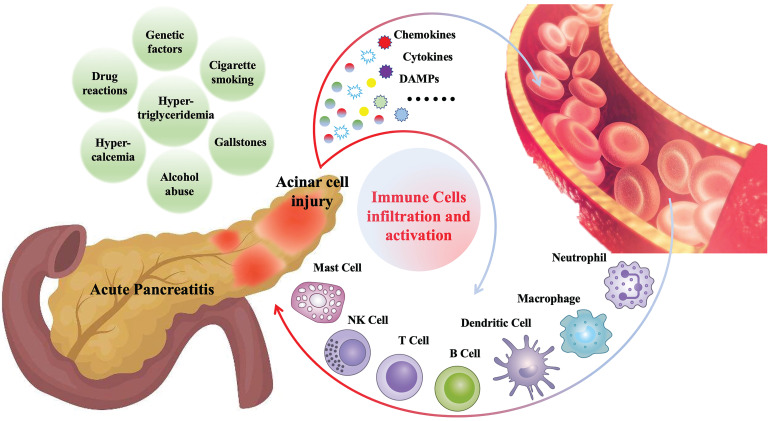
Gallstones, alcohol abuse, hypertriglyceridemia, hypercalcemia, cigarette smoking, drug reactions and genetic factors are the common causes of AP. During AP, injured pancreatic acinar cells release a series of pro-inflammatory mediators (cytokines, chemokines, DAMPs, etc.), which promote the recruitment and activation of immune cells, including innate immune cells such as neutrophils, macrophages, dendritic cells, mast cells, NK cells, and adaptive immune cells such as T cells and B cells.

Various immune cells begin to infiltrate the pancreas within minutes after the onset of AP and are closely related to the severity and prognosis of AP 8, 13. These infiltrated immune cells include innate immune cells, such as macrophages 14, neutrophils 15, dendritic cells 16, mast cells 17, natural killer cells (NK cells) 18, as well as adaptive immune cells, such as T lymphocytes 19, B lymphocytes 19. Cells of the innate immune system like neutrophil and macrophage represent the majority of infiltrating cells. Generally, immune cell infiltration, as a defense mechanism, is beneficial for disease recovery. However, in some cases, the pathogenic factors cannot be cleared in the short term, and the immune cells related inflammatory response will persist and amplify, which may further aggravate pancreatic damage and contribute to systemic inflammation 20, 21. **Figure 1** is a summary of common causes of AP and mechanisms of immune cell infiltration during AP.

In this review, an overview of different populations of innate immune cells and their role during AP will be discussed, with a special focus on neutrophils and macrophages.

## Neutrophils

### Neutrophils and inflammation

Neutrophils, a kind of polymorphonuclear cells with the rod or lobulated nuclei, originating from bone marrow hematopoietic stem cells, accounting for 60-70% of the circulating leucocytes and among the first cells recruited to the inflammatory site 22. Neutrophils have long been recognized as potent pathogen scavengers, whose cytoplasmic granules contain bactericidal substances such as myeloperoxidase (MPO), acid phosphatase, alkaline phosphatase, lysozyme, and defensins. However, In recent years, increasing evidence suggests that neutrophils also play an important role in sterile inflammation, they are required to clear cell debris produced in the process of tissue damage to restore cell homeostasis 22-25. During an infection or sterile inflammation, damaged tissue cells may produce chemokines, such as CXC chemokine ligands 1 (CXCL1), CXC chemokine ligands 2 (CXCL2), and CXC chemokine ligands 8 (CXCL8) 22, 26, which can activate neutrophils. Then, under the interaction of a series of integrins including very late antigen-4 (VLA-4), macrophage-1 antigen (Mac-1), lymphocyte function-associated antigen-1 (LFA-1) 26, selectin (P-selectin, E-selectin) 27 and adhesion molecule such as intercellular adhesion molecule-1 (ICAM-1) 28, neutrophils ultimately reach the inflammatory site. Besides, PAMPs released by pathogens and DAMPs released by damaged tissue cells can also directly activate PRRs on neutrophils, and then recruit neutrophils to the inflammatory site 29.

It is generally accepted that the clearance of pathogens and cell fragments is accomplished by phagocytosis and degranulation, two classical functions of neutrophils. However, in 2004, Brinkmann first reported that the production of neutrophil extracellular traps (NETs) also enables neutrophils to do this 30. NETs are weblike structures released by activated neutrophils into extracellular space, with DNA as its scaffold, which is decorated with histones, MPO, neutrophil elastase (NE), cathepsin G, calreticulin, protease 3, HMGB1, etc. 30, 31. Since the formation of NETs is usually accompanied by the death of neutrophils, this process is also called NETosis 32. NETs were initially thought to be a mechanism by which neutrophils clear pathogens, for example, the replication of staphylococcus aureus, salmonella typhimurium, streptococcus pneumoniae, and shigella flexneri can be inhibited by NETs 30, 33-35. But its critical role in sterile inflammation has also been revealed in recent years, such as deep venous thrombosis 36, lung cancer 37, arthritis 38, AP 25, 39, 40, endogenous particulate-related injury 41, glioma 31, liver ischemia-reperfusion injury 42, ischemic stroke 43, etc.

In summary, neutrophils are important effector cells in the maintenance of immune surveillance and their clearance of pathogens effectively limit the spread of infection and the development of sepsis 44. Also, neutrophils can be recruited to the sterile inflammatory sites, clearing necrotic tissue and cells, and promoting tissue repair 45. However, the infiltration of neutrophils is a double-edged sword, lack of resolution or persistent local inflammation may lead to a more aggressive neutrophil response 46 characterized by the destruction of normal tissue and uncontrolled systemic inflammation.

### Neutrophils infiltration in the pathogenesis of AP and therapy

**Figure 2:**
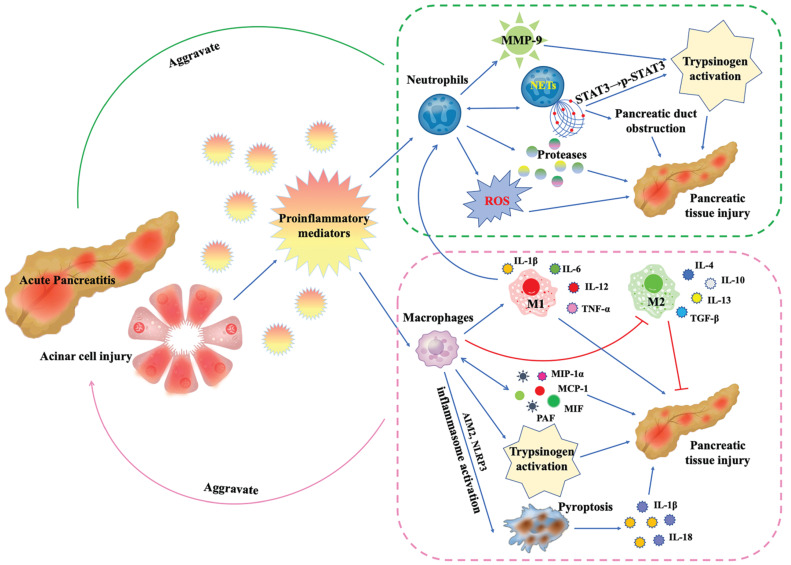
During AP, damaged pancreatic acinar cells release proinflammatory mediators, leading to the recruitment, activation and functional changes of neutrophils and macrophages: (1) Neutrophils release proteases and ROS, which directly damage pancreatic tissue.The released MMP-9 is a potent trypsinogen activator. In addition, activated neutrophils can form NETs, which promotes trypsinogen activation by phosphorylating STAT3 and leads to pancreatic duct obstruction by forming aggregates. More critically, the formation of NETs will in turn promote more neutrophil infiltration. (2) Macrophages secrete inflammatory mediators MIP-1α, MCP-1, MIF and PAF to directly damage pancreatic tissue, and in turn promote more macrophage infiltration. In AP, inflammatory mediator microenvironment promotes macrophage M1 polarization and inhibits M2 polarization. M1 macrophages secrete IL-1β, IL-6, IL-12 and TNF-α to mediate pancreatic inflammation and injury. Like neutrophils, macrophages are also involved in trypsinogen activation. In addition, AIM2 or NLRP3 inflammasome activation within macrophages triggers macrophage pyroptosis, leading to the rupture of plasma membrane, maturation and release of IL-1β and IL-18, aggravating inflammation and pancreatic injury.

A substantial number of studies have suggested that neutrophils are involved in the pathogenesis of AP 25, 27, 28, 39, 40, 47-76 (**Figure 2**). Measurements of MPO levels in serum 27 or pancreatic tissue 49, 54, 61 can reflect the number and activity of infiltrating neutrophils. The commonly used methods include commercial detection kits 61, enzyme-linked immunosorbent assay (ELISA) 27, 49, and immunofluorescence 54. In addition, for mice and rats AP models, MPO-positive cells, or Ly6G-positive cells in pancreatic tissues, which are commonly used markers of neutrophils, can also be detected by immunohistochemistry 21, 28, 66.

Intrapancreatic activation of trypsinogen is a key event in AP, which leads to acinar cell injury 69. During the development of AP, neutrophils infiltrating the pancreas play a critical role in pathologic activation of trypsinogen and modulation of inflammation 69, 74. The depletion of neutrophils by the administration of antineutrophil serum 69 or anti-Gr-1 antibody 74 exhibited a significant reduction in intrapancreatic trypsinogen activation, trypsin-activation peptide (TAP) level, and tissue damage. Neutrophil-derived matrix metalloproteinase-9 (MMP-9) is also a potent activator of trypsinogen activation in pancreatic acinar cells during AP 68. The accumulation of reactive oxygen species (ROS) and related oxidative stress are also involved in the initiation of AP 71. The high nicotinamide adenine dinucleotide phosphate (NADPH) oxidase activity of neutrophils makes them the main source of ROS in AP, mediating oxidative damage to the pancreas and promoting trypsinogen activation 69. In addition, proteases released by neutrophils, such as elastase, can also contribute to tissue damage during AP 47, 58, while the deficiency of Cathepsin C alleviates AP through the reduction of neutrophil elastase activation 50. Based on the above evidence, we can conclude that neutrophils are closely involved in the initiation and development of AP and are associated with the severity. Therefore, more and more scientists are devoted to exploring the mechanism of neutrophil infiltration in AP and looking for intervention targets.

Genetic or pharmacological inhibition of some specific gene targets, or administration of some medication, can alleviate AP through reducing neutrophils infiltration or NETs formation. The nuclear factor of activated T cells 3 (NFATc3) is an important downstream target of calcineurin and is up-regulated in AP. Knockdown of NFATc3 or the administration of the NFAT inhibitor A-285222 both significantly reduced neutrophil infiltration and pathological damage in the pancreas and lung in AP 70. Inhibition of LFA-1, one of the integrins, by gene knockout or anti-LFA-1 antibody significantly reduced neutrophil recruitment and activation in AP, thereby reducing pancreatic injury 65. Galectin-3, as a member of the galectins family, plays an important pro-inflammatory role in inflammatory diseases. The deletion of galectin-3 decreases early neutrophils influx in AP 51. Inhibition of Ras signaling by farnesylthiosalicylic acid (FTS) can also attenuate SAP by reducing neutrophil infiltration 63. Loss of tissue nonspecific alkaline phosphatase (TNAP) 56 or receptor-activity-modifying-protein-1 (RAMP1) 67 expression markedly increase neutrophil infiltration and tissue damage in AP, thus maintaining their expression may be a therapeutic strategy. Several widely studied or novel medications, including 4-phenyl butyric acid (4-PBA) 62, lactose 64, pentoxifylline 72, zileuton 27, desoxo-narchinol-A 59, Evasin-3 76, biomimetic carbon monoxide delivery 48 and Emodin 53, can abrogate pancreatic neutrophil infiltration and associated inflammation in AP.

### NETs and AP

As a special functional state of neutrophils, NETs participate in multiple processes of AP pathogenesis. A recent study has shown that NETs are also involved in the activation of trypsinogen 25. Histones 2A, 2B, 3, and 4, the major protein of NETs structure, are the key components for increasing trypsin activity, which promotes the activation of trypsin by phosphorylating Signal Transducer and Activator of Transcription 3 (STAT3) in pancreatic acinar cells 25. Interestingly, the formation of NETs in the pancreas and lung during AP can recruit more infiltrating neutrophils, which in turn aggravate tissue damage 25. Moreover, NETs can directly activate neutrophils, promoting their expression of Mac-1 as well as the generation of ROS and MMP-9 25. NETs can also regulate systemic inflammation by upregulating HMGB1, CXCL2, and IL-6 25. Obstruction of the pancreatic duct can also lead to AP and determine its severity 52. During AP, the pancreatic juice is a strong instigator of infiltrating neutrophil chromatin extrusion and thereby lead to the formation of macroscopic NETs aggregates, which may occlude pancreatic duct and drive pancreatic inflammation, inhibition of NETs formation can mitigate AP 57. For the detection of NETs formation in AP, cell-free DNA (cfDNA) 55, MPO-DNA complex 55, or histone-DNA complex 39, 75 in blood samples can be measured by commercial kits. Besides, citrullinated histone H3 (CitH3) 39, 75 in pancreatic samples can be measured by western blot.

Peptidyl arginine deiminase 4 (PAD4) and c-Abelson (c-Abl) kinase are responsible for regulating the formation of NETs 39, 60. Pharmacologically inhibition of PAD4 by Cl-amidine, a pan-PAD inhibitor, or inhibition of c-Abl by GZD824, a c-Abl kinase inhibitor, can reduce NETs formation and tissue Injury in SAP 39, 75. The autophagy inhibitor chloroquine (CQ) also inhibits NETs formation, thus reduces the severity of AP 55. Recent studies have shown that platelets also have a role in the regulation of neutrophils recruitment and NETs formation. Activated platelets induce neutrophils recruitment by secreting CXCL4 and promote NETs formation by producing inositol hexakisphosphate kinase 1 (IP6K1). Depletion of platelets, inhibition of IP6K1 by TNP, or inhibition of CXCL4 can alleviate neutrophil infiltration, NETs formation, and tissue damage in AP 40, 73.

## Macrophages

### Macrophages and inflammation

Macrophages include tissue-resident macrophages and migrating macrophages. Evidence has shown that tissue-resident macrophages are derived from the yolk sac and seeded before birth, which has self-renewal ability 77, 78. The names of tissue-resident macrophages vary among different organs, for example, Kupfer cells in the liver, microglia in the brain, and osteoclasts in bone. Migrating macrophages are derived from bone marrow stem cells. Stimulated by the multi-colony stimulating factor (multi-SCF) and macrophage colony-stimulating factor (GM-CSF), bone marrow stem cells develop into granulocyte-monocyte progenitor cells, then differentiate into promonocytes and enter the blood, and finally mature into monocytes, accounting for 3-8% of the peripheral blood leukocytes. Monocytes exhibit a short half-life of 20 hours 79, then migrate to tissues and organs and differentiate into macrophages.

Macrophages can clear pathogens, tissue debris, necrotic and apoptotic cells through their effective phagocytosis, playing an important role in infectious and sterile inflammation 80. They are not only an important component of innate immunity but also involve in regulating adaptive immune response as antigen-presenting cells (APCs) 81. High plasticity is a hallmark of macrophages. Macrophage polarization is a phenotypical and functional change in response to microenvironment variations 80. In the process of infection and sterile inflammation, tissue-resident macrophages play an important role in detecting PAMPs released by pathogens and DAMPs released by damaged cells and then secrete a variety of pro-inflammatory mediators to recruit more tissue-resident macrophages and circulating monocytes to the inflammatory sites 82, where the cytokine microenvironment dominates the orientation of macrophage polarization 83. Macrophages commonly exist in two distinct subsets, including classically activated (M1) and alternatively activated (M2) macrophages, the former are generally polarized by Th1 cytokines IFN-γ, TNF-α and act in a pro-inflammatory manner by secreting interleukin (IL)-1β, IL-6, IL-12 and tumor necrosis factor (TNF-α), while the latter is induced by Th2 cytokines IL-4, IL-13 and play an anti-inflammatory, immunoregulatory and pro-fibrotic role by secreting IL-4, IL-10, IL-13 and transforming growth factor-β (TGF-β) 80, 82, 83. It is worth mentioning that M2 macrophages can be further subdivided in M2a, M2b, M2c, and M2d based on the applied stimuli and the resultant transcriptional changes 84. M2a macrophages, also known as wound healing macrophages, promote tissue repair and regeneration by secreting the profibrotic factor TGF-β 85. M2b is a regulatory macrophage that can secrete both pro-inflammatory and anti-inflammatory mediators 85. M2c macrophages are anti-inflammatory macrophages, mainly secrete anti-inflammatory cytokine IL-10, and can efficiently phagocytose apoptotic cells 85. M2d macrophages represent a novel M2 subset that is also known as tumor-associated macrophages (TAMs) and mainly plays a role in angiogenesis and metastasis of tumors 86. Macrophage polarization imbalance, especially the type in which the ratio of M1 to M2 is significantly increased, is one of the mechanisms underlying the aggravation of many inflammatory diseases, including autoimmune myocarditis 87, inflammatory root resorption 88, brain ischemic injury 89, acute lung injury 90, AP 91, intestinal ischemia/reperfusion injury 92 and so on. M1 macrophages exacerbate tissue damage and impair tissue regeneration by secreting a large quantity of pro-inflammatory cytokines to induce inflammatory cascade, triggering ROS-mediated inflammation through activation of NADPH oxidase 82, as well as activating neutrophils 93.

Since the initial description of NETs appeared in 2004 30, it is now known that macrophages are also able to produce “extracellular trap” structures through METosis 94. METs function to immobilize and kill some pathogenic microorganisms, but may also play a role in disease pathology 95-97. Inadequate resolution and degradation of METs, as well as prolonged exposure of self-antigens comprising METs may contribute to inflammation and autoimmune diseases 98, such as autoimmune arthritis 95, atherothrombosis 96, rhabdomyolysis-induced acute kidney injury 97.

In recent years, the critical role of macrophage pyroptosis in inflammatory diseases has been revealed. Pyroptosis is a rapid, proinflammatory, programmed cell necrosis mediated by gasdermin family proteins (GSDMA, GSDMB, GSDMC, GSDMD, GSDME, DFNB59), characterized by cellular swelling, membrane disruption, and release of proinflammatory cellular contents, such as IL-1β, IL-18 and HMGB1 99, 100. Macrophage pyroptosis is involved in and aggravates many inflammatory diseases, such as knee osteoarthritis 101, sepsis 102, liver ischemia-reperfusion injury 103, multiple sclerosis 104, retinal detachment-induced photoreceptor cell death 105, while inhibition of macrophage pyroptosis can significantly reduce the severity of the disease 101-105.

### Macrophages infiltration in the pathogenesis of AP and therapy

Like neutrophils, macrophages are also the main innate immune cells involved in the pathogenesis of AP, infiltrating the inflamed pancreas in the early phase of AP and promoting the development of AP 19-21, 48, 91, 106-128 (**Figure 2**). Detection of F4/80 positive cells or CD68 positive cells in the pancreas by immunofluorescence or immunohistochemistry is the main method to detect pancreatic infiltrating macrophages in mice 67, 106, 129.

Damaged pancreatic acinar cells recruit macrophages by releasing DAMPs and proinflammatory mediators, such as monocyte chemo attractant protein-1 (MCP-1, also known as CCL2), TNF-α, etc. 107-109. In addition, pancreatic acinar cell death-derived DNA, pancreatic elastase, lipase, pancreatitis-associated ascites, and even lipid extracts from ascites can activate infiltrating macrophages 110-112, and the degree of macrophage activation is positively correlated with the severity of AP 113, 114. These infiltrating macrophages produce proinflammatory mediators including TNF-α, IL-1β, IL-6, IL-18, MCP-1, platelet-activating factor (PAF), macrophage inflammatory protein (MIP)-1α and macrophage migration inhibitory factor (MIF), macrophage inflammatory protein-2 (MIP-2) 115-118, thus, more macrophages are recruited, triggering and further amplifying local inflammation, aggravating tissue damage. The depletion of macrophages through the administration of clodronate liposomes significantly reduced disease severity in caerulein-induced AP model 20, suggesting an important role of macrophages in the pathogenesis of AP. Construction of caerulein-induced AP model in CCR2 (receptor of CCL2) deficient mice showed that infiltrating macrophages in the pancreas were significantly reduced and tissue damage was significantly mitigated 107. Targeting macrophage associated inflammatory mediators in AP also achieves a good therapeutic effect. Treatment with bindarit, which blocks the synthesis of MCP-1, or injection of a plasmid expression vector containing a dominant-negative mutant MCP-1 gene (mMCP-1), significantly reduced the severity of AP 117, 119. Prophylactic administration of anti-MIF antibody significantly improved the survival rate of mice and rat model of SAP 116. Pretreatment with ISO-1, an inhibitor of MIF, also notably attenuated the severity of AP 120. Blockade of MIP-2 by anti-MIP-2 antibody significantly reduced the infiltration of inflammatory cells in the pancreas and lung, and reduced tissue damage in AP 118. For PAF, the administration of PAF receptor antagonists (PAF-RAs) could significantly reduce local and systemic events after AP 130.

### Macrophages polarization and AP

With the polarization of macrophages, M1 and M2 subtypes express unique markers, such as CD40, iNOS, TNF-α, IL-1β and IL-6 of M1 macrophages, Arg-1, Clec10, Mrc1 and CD206 of M2 macrophages 91, 131, which are the common markers for detecting M1 and M2 macrophages.

Since the polarization status of macrophages is related to the progression and severity of AP, many researchers have focused on the therapeutic effect of regulating the polarization status of macrophages on AP 48, 91, 121, 122. It is generally accepted that abdominal paracentesis drainage (APD) can alleviate AP. A recent study revealed that pancreatitis-associated ascitic fluids (PAAF) can promote macrophage M1 polarization, and the therapeutic effect of APD is partly achieved by promoting macrophage M2 polarization and inhibiting M1 polarization 121. Adipose stem cells (ASCs) are characterized by significant immunomodulatory and regenerative ability. The proportion of anti-inflammatory M2 phenotype in pancreatic macrophages was significantly increased by the infusion of ASCs into the tail vein of SAP mice model 122. The infusion of carbon monoxide-bound hemoglobin vesicles (CO-HbV), as a nanomedicine modality, polarized macrophages toward an M2 phenotype and in the pancreas and mitigated AP 48. Inducing SAP in LysM^+/Cre^DRD2^fl/fl^ mice exhibited more M1 polarization of pancreatic macrophages and stronger inflammatory response, suggesting that myeloid-specific dopamine D2 receptor (DRD2) could protect AP by inhibiting M1 polarization 91. Inhibition of macrophage M1 polarization in the pancreas and liver can also reduce AP-related liver injury and lung injury 123, 124.

### Macrophages pyroptosis and AP

As a kind of potent pro-inflammatory cell death, pyroptosis plays an important role in local and systemic inflammation in AP. Genetic deletion of Nod-like receptor family pyrin domain-containing 3 (NLRP3), absent in melanoma-2 (AIM2), apoptosis-associated speck-like protein containing caspase recruitment domains (ASC), or Caspase-1, which are the components of the inflammasome, markedly reduced the severity of AP 109, 125, 126. MCC950, an NLRP3 inhibitor, can also reduce the severity of AP 125. Besides, the administration of lactate can alleviate SAP by inhibiting NLRP3 inflammasome activation in macrophages 127. More importantly, studies have shown that pyroptosis occurs mainly in infiltrating macrophages, but not in pancreatic acinar cells during AP 109, 125. A recent study revealed the mechanism of pyroptosis in infiltrating macrophages. The infiltrating macrophages were found to phagocytose zymogen-containing vesicles from injured acinar cells and activate trypsinogen intracellularly, which acts as DAMPs and induces NLRP3 inflammasome activation within macrophages. Inhibition of trypsinogen activation within macrophages reduced pro-inflammatory cytokine secretion and mitigated AP 21. Interestingly, a recent study suggested that the inflamed pancreas releases exosomes containing various signaling molecules which leads to SAP associated lung injury through NLRP3 inflammasome activation and subsequent pyroptosis in alveolar macrophages (AMs). Inhibition of exosome release or uptake by GW4869 or Enoxaparin significantly reduced AMs pyroptosis and thus alleviated SAP-induced lung injury 128. These studies indicate that targeting pyroptosis of infiltrating macrophages may be a novel and effective strategy for the treatment of AP.

## Other innate immune cells in AP

### Dendritic cells

Dendritic cells (DCs) originate from bone marrow hematopoietic stem cells and are the most powerful antigen-presenting cells. DCs can phagocytose and clear pathogens as well as harmful antigens to exert innate immune functions. Also, DCs can activate T and B cells through antigen presentation, and regulate immune responses by secreting various cytokines. Therefore, DCs are the link between innate and adaptive immunity 132.

In AP, infiltrating DCs seem to play a protective role in some cases 16, 133, 134, while in others, they aggravate the severity of AP 135-137. DCs increased 100-fold in the pancreas of caerulein- or L-arginine-induced SAP mouse models, presenting the phenotype of MHC II+CD11c+, secreting IL-6, MCP-1, and TNF-a. Interestingly, rather than inducing an organ-destructive inflammation, DCs were required for promoting pancreatic viability. SAP mice that were depleted of DCs by diphtheria toxin died from severe acinar cell death within 4 days 16. In type B coxsackieviruses (CVB)-induced AP, the inflamed pancreas secretes CCL17, which binds to CCR4 on DCs and recruits them to the site of inflammation. The infiltrating DCs triggers Th1 immune responses, thereby reducing viral load and tissue damage. CCR4 knockout resulted in a reduced recruitment of DCs, elevated viral load, and increased severity of AP 133. Furthermore, DCs limit neutrophils infiltration and tissue damage by expressing dendritic cell natural killer lectin group receptor-1 (DNGR-1) encoded by the gene Clec9a as a feedback mechanism. Knockout of DNGR-1or inhibition of DNGR-1 by DNGR-1-blocking antibody significantly aggravated tissue damage 134. However, increasing DCs activity by MyD88 inhibition exacerbated pancreatic inflammation 137. Supplementation with Clostridium butyricum, a probiotic, can alleviate tissue damage and inflammation by reducing the infiltration of DCs in AP 135. A recent study reported an interesting phenomenon that in AP, pancreatic acinar cells undergo acinar-to-dendritic cell transition, which in turn promotes the differentiation of naive CD4+T cells into CD4+/IFN-γ+Th1 and CD4+/IL-17A+Th17 cells, thereby aggravating local inflammation and tissue damage. The administration of mTOR inhibitor rapamycin or Myc inhibitor 10058-F4 can inhibit acinar-to-dendritic cell transition, thus reducing the severity of AP 136. In summary, DCs play both pro- and anti-inflammatory roles in AP, subsequent studies are needed to further uncover their roles in AP.

### Mast cells

Mast cells originate from bone marrow stem cells. Activated mast cells release cytoplasmic granules including histamine, serotonin, protease, cytokines, and chemokines in a process known as degranulation. Mast cells are best well-known for their expression of high-affinity IgE receptors and involvement in IgE-mediated type I hypersensitivity. However, recent data indicate that mast cells are also pivotal players in innate and adaptive immune responses 138. For example, abnormal accumulation and activation of mast cells are present in AP 17, 139-143.

Elevated mast cell count and mast cell degranulation were observed in the pancreas during AP 17, 139. Treatment with ketotifen, a mast cell stabilizer, significantly reduced pancreatic tissue damage 139. Intraperitoneally injection of mast cell inhibitor cromolyn significantly reduced inflammation in the pancreas and lung 17. Administration of sodium cromoglycate (SCG), another mast cell stabilizer, decreased the release of histamine and reduced plasma exudation in the pancreas, colon, and lungs, suggesting that mast cell activation is involved in the development of endothelial dysfunction in the pancreas and other distant organs in AP, which may underlie MODS 142. In addition, the inflamed pancreas expressed and released IL-33 during AP. As an ST2 ligand, IL-33 reduced the activation of mast cells after binding with ST2 on mast cells, which is a protective mechanism for AP. ST2 knockout leads to more severe AP 143. The activated infiltrating mast cells can also secrete IL-33 and histamine, which drives pancreatic inflammation 140. Scopoletin reduced the severity of pancreatic and associated lung injury in AP by reducing mast cell activation and corresponding levels of IL-33 141. Therefore, the mast cells may hold promise as a treatment target in AP.

### NK cells

NK cells originated from bone marrow lymphoid stem cells, accounting for 5%-15% of peripheral blood lymphocytes. NK cells can kill tumor cells or virus-infected cells directly or through antibody-dependent cell-mediated cytotoxicity (ADCC). Besides, they are also involved in maintaining immune homeostasis and regulating inflammation 144. So far, studies on NK cells in AP are limited. Several studies have reported that NK cells infiltrate the pancreas during AP 18, 145. In the adenoviral vector-mediated AP model, NK cells infiltrated the inflamed pancreas from the second day, reaching a maximum on the fourth day, and persisting until 28 days 18, however, their role in the pathogenesis of AP is not clear. More studies focused on changes in NK cells in peripheral blood. Studies have shown that peripheral blood NK cell counts in patients with AP were lower than those in healthy controls 146, 147. The activity of NK cells and ADCC decreased in some SAP patients 148. Compared with MAP, patients with SAP experienced a significant decrease in the number of peripheral blood NK cells in the early phase and persisted for 30 days 147, 149. The immunosuppressive state of peripheral leukocyte and NK cell depletion is thought to be responsible for the infectious complications of AP 148, 149. More studies are needed to explore the exact role of NK cells in AP.

## Conclusions and future prospects

AP begins with abnormal activation of trypsinogen and self-digestion. Some patients experience a transition from pancreatic local inflammation to systemic inflammation even MODS. However, the current understanding of the pathogenesis of AP is still far from complete, limiting our treatment options. With the advances of research in the past 20 years, the role of innate immune cells, including neutrophils, macrophages, dendritic cells, mast cells, in the inflammatory storm of AP has become increasingly prominent, however, reports on the role of infiltrating eosinophils and basophils in AP are lacking. While promoting pancreatic injury, these innate immune cells also interact with each other and adaptive immune cells to form a huge regulatory network. A substantial of studies have shown that the intervention of immune cells through pharmacological and genetic methods has indeed alleviated AP, bringing hope for the treatment of AP. Immunotherapy, as an emerging therapy, is very promising. However, immunotherapy is the future since the underlying immunological mechanisms of AP are rather complex, a lot of works are needed to clarify the regulatory network. Unlike neoplastic diseases, the acquisition of tissue samples from patients with AP is difficult, which limits the relevant scientific research to some extent. Therefore, direct evidence of immunological alternations of AP in the human pancreas is lacking. At present, the widely used caerulein-induced mice AP model and the sodium taurocholate-induced rat AP model cannot fully correspond to the clinical AP with various etiological backgrounds. Therefore, according to the current evidence, it is difficult to answer whether AP of different etiological backgrounds shares the same immunological characteristics. This requires further exploration at the patients' tissue level or in animal models of more types of AP before conclusions can be drawn. In summary, we believe that for the treatment of AP, immunotherapy is a powerful means in the future, which has high clinical transformation value. But before that, we still have a lot of work to do.

## Abbreviations

AP: acute pancreatitis
MAP: mild acute pancreatitis
SAP: severe acute pancreatitis
SIRS: systemic inflammatory response syndrome
MODS: multiple organ dysfunction syndromes
MOF: multiple organ failure
PAMPs: pathogen-associated molecular patterns
DAMPs: danger-associated molecular patterns
HMGB1: high-mobility group box protein 1
PRRs: pattern recognition receptors
NK cells: natural killer cells
MPO: myeloperoxidase
NETs: neutrophil extracellular traps
NE: neutrophil elastase
ELISA: enzyme-linked immunosorbent assay
TAP: trypsin-activation peptide
MMP-9: matrix metalloproteinase-9
ROS: reactive oxygen species
NADPH: nicotinamide adenine dinucleotide phosphate
NFATc3: nuclear factor of activated T cells 3
FTS: farnesylthiosalicylic acid
TNAP: tissue nonspecific alkaline phosphatase
RAMP1: receptor-activity-modifying-protein-1
4-PBA: 4-phenyl butyric acid
STAT3: Signal Transducer and Activator of Transcription 3
cfDNA: cell-free DNA
CitH3: citrullinated histone H3
PAD4: Peptidyl arginine deiminase 4
c-Abl: c-Abelson
CQ: chloroquine
IP6K1: inositol hexakisphosphate kinase 1
multi-SCF: multi-colony stimulating factor
GM-CSF: macrophage colony-stimulating factor
APCs: antigen-presenting cells
TNF-α: tumor necrosis factor-α
TGF-β: transforming growth factor-β
MCP-1: monocyte chemo attractant protein-1
PAF: platelet-activating factor
MIF: macrophage migration inhibitory factor
MIP: macrophage inflammatory protein
mMCP-1: mutant MCP-1 gene
PAF-RAs: PAF receptor antagonists
APD: abdominal paracentesis drainage
PAAF: pancreatitis-associated ascitic fluids
ASCs: adipose stem cells
CO-HbV: carbon monoxide-bound hemoglobin vesicles
DRD2: dopamine D2 receptor
NLRP3: Nod-like receptor family pyrin domain-containing 3
AIM2: absent in melanoma-2
ASC: apoptosis-associated speck-like protein containing caspase recruitment domains
AMs: alveolar macrophages
DCs: dendritic cells
CVB: type B coxsackieviruses
DNGR-1: dendritic cell natural killer lectin group receptor-1
SCG: sodium cromoglycate
ADCC: antibody-dependent cell-mediated cytotoxicity
TAMs: tumor-associated macrophages
CXCL1/CXCL2/CXCL8: CXC chemokine ligands 1/2/8
VLA-4: very late antigen-4
Mac-1: macrophage-1 antigen
LFA-1: lymphocyte function-associated antigen-1
ICAM-1: intercellular adhesion molecule-1
